# Depressive disorder and gastrointestinal dysfunction after myocardial infarct are associated with abnormal tryptophan-5-hydroxytryptamine metabolism in rats

**DOI:** 10.1371/journal.pone.0172339

**Published:** 2017-02-17

**Authors:** Xiaofang Lu, Yuefen Wang, Chunyan Liu, Yangang Wang

**Affiliations:** 1 Digestive Disease Center, Beijing Hospital of Traditional Chinese Medicine Affiliated to Capital Medical University, Beijing, China; 2 Department of Nephropathy, Beijing Hospital of Traditional Chinese Medicine Affiliated to Capital Medical University, Beijing, China; 3 Department of Rheumatology, The Third Hospital Affiliated to Hebei Medical University, Shijiazhuang, Hebei, China; 4 Department of Gastroenterology, Hebei Provincial Hospital of Traditional Chinese Medicine, Shijiazhuang, Hebei, China; Technion Israel Institute of Technology, ISRAEL

## Abstract

In this study, we investigated the relationship between tryptophan-5-hydroxytryptamine metabolism, depressive disorder, and gastrointestinal dysfunction in rats after myocardial infarction. Our goal was to elucidate the physiopathologic bases of somatic/psychiatric depression symptoms after myocardial infarction. A myocardial infarction model was established by permanent occlusion of the left anterior descending coronary artery. Depression-like behavior was evaluated using the sucrose preference test, open field test, and forced swim test. Gastric retention and intestinal transit were detected using the carbon powder labeling method. Immunohistochemical staining was used to detect indoleamine 2,3-dioxygenase expression in the hippocampus and ileum. High-performance liquid chromatography with fluorescence and ultraviolet detection determined the levels of 5-hydroxytryptamine, its precursor tryptophan, and its metabolite 5-hydroxyindoleacetic acid in the hippocampus, distal ileum, and peripheral blood. All data were analyzed using one-way analyses of variance. Three weeks after arterial occlusion, rats in the model group began to exhibit depression-like symptoms. For example, the rate of sucrose consumption was reduced, the total and central distance traveled in the open field test were reduced, and immobility time was increased, while swimming, struggling and latency to immobility were decreased in the forced swim test. Moreover, the gastric retention rate and gastrointestinal transit rate were increased in the model group. Expression of indoleamine 2,3-dioxygenase was increased in the hippocampus and ileum, whereas 5-hydroxytryptamine metabolism was decreased, resulting in lower 5-hydroxytryptamine and 5-hydroxyindoleacetic acid levels in the hippocampus and higher levels in the ileum. Depressive disorder and gastrointestinal dysfunction after myocardial infarction involve abnormal tryptophan-5-hydroxytryptamine metabolism, which may explain the somatic, cognitive, and psychiatric symptoms of depression commonly observed after myocardial infarction. Peripheral 5-hydroxytryptamine is an important substance in the gut-brain axis, and its abnormal metabolism is a critical finding after myocardial infarct.

## Introduction

Depression is a common psychiatric symptom of myocardial infarction [1] and is an independent risk factor for disease prognosis. Besides this, myocardial infarction has been previously reported to have an effect on intestinal barrier integrity [2] and somatic psychiatric symptoms (e.g., gastrointestinal symptoms, fatigue, poor appetite, and nausea) that also reflect the prognosis of the disease. However, no previous study has investigated the association between the mechanisms of somatic and psychiatric symptoms after myocardial infarction. A better understanding of depression and gastrointestinal disorders after myocardial infarction will increase their utility as prognostic indicators and improve the therapeutic approach to myocardial infarction.

5-Hydroxytryptamine (5-HT), also known as serotonin, is an abundant neurotransmitter in the central and peripheral nervous systems, especially the enteric nervous system, and plays crucial roles in both depression and gastrointestinal function. Low metabolism of hippocampal 5-HT leads to abnormalities in brain regions associated with learning, memory, and emotion. It functions through the binding of its receptors, including the G-protein coupled 5-HT_1A_ receptor, which is a predominant contributor to the regulation of emotion in the hippocampal CA1 region and the dentate gyrus. Stimulation of the 5-HT_1A_ receptor inhibits receptor-G protein-cyclic adenosine monophosphate or mitogen-activated protein kinase pathways in order to affect hippocampal synaptic plasticity and neural regeneration [3]. Accumulating evidence indicates that hippocampal function is closely associated with gastrointestinal symptoms, and hippocampal signals are active in irritable bowel syndrome patients [4]. Further, animal studies have shown that the levels and metabolism of 5-HT in the hippocampus are low in rats with post-infectious irritable bowel syndrome [5]. These data suggest that the hippocampus is involved in the regulation of gastrointestinal function.

Here, we hypothesize that depression and gastrointestinal disorders after myocardial infarction are associated with central and enteric 5-HT dysfunction. Using a rat model of myocardial infarction, we observed depression-like behavior and gastrointestinal disorders concurrent with changes in tryptophan-to-5-HT metabolism in the hippocampus and intestinal tract, which may provide novel targets for the prognosis and biological basis of somatic/psychiatric depression symptoms after myocardial infarction.

## Materials and methods

### Ethics statement

The use of experimental animals was in strict accordance with the Regulations for the Administration of Affairs Concerning Experimental Animals and was approved by the local ethical review committee on animal care of the Beijing University of Chinese Medicine (2012BZHYLL01034).

### Animals

A total of 35 male specific pathogen free Wistar rats, aged 8 weeks and weighing 250–280 g, were purchased from Beijing Vital River Laboratory Animal Technology Co., Ltd., Beijing, China (license number SYXK (Beijing) 2012–0001). All animals were housed in the Experimental Animal Center at the Beijing University of Chinese Medicine under conditions of constant temperature (18–24°C) and relative humidity (45–65%) on a 12-hour light/dark cycle (lights on 7:00–19:00). Animals were allowed free access to food and water.

### Experimental design

The 35 rats were randomly divided into 3 groups: an acute myocardial infarction model group (model group; n = 15), a sham operation group (sham group; n = 12), and a normal group (n = 8). Rats were evaluated with surface electrocardiography preoperatively and postoperatively. The myocardial infarction models were successfully established on post-operative day 21, after which the rats were subjected to the open-field test on post-operative days 22–24, forced swim testing on days 25–26, and sucrose preference testing on day 28.

### Harvesting of tissue

Rats were anesthetized with 10% chloral hydrate (0.4 mL/kg), and 4 mL abdominal aortic blood was collected and centrifuged in heparin tubes. Supernatants were stored at -80°C until use. Rats were sacrificed by decapitation and the stomach and small intestine were isolated for the determination of the gastric residual rate and the small intestine propulsion rate. Cardiac, right hippocampal, and 2 cm length of ileal tissues were rinsed with ice-cold saline, and subsequently about 25 mg (about one half) right hippocampus and a 1 cm length of the ileum were preserved at -80°C. The remaining tissues were fixed with 4% paraformaldehyde.

### Establishing myocardial infarction models

The myocardial infarction model was produced according to a non-ventilator-assisted method developed by Trueblood et al. [6] with minor modifications. Briefly, Wistar rats were anesthetized with 10% chloral hydrate (0.4 mL/kg) and positioned overhead on the operating table. A baseline surface electrocardiogram was recorded using a biological signaling quantitative analysis system (sp-2006, Beijing Softron Biotechnology Ltd., Beijing, China). The surgical site was shaved and disinfected with iodine. A median incision was made and the intercostal muscle was stripped with a hemostat. A thoracotomy was performed between the 3^rd^ and 4^th^ left ribs to expose the heart. Subsequently, the left anterior descending coronary artery was ligated using a 6–0 non-invasive thread 2–3 mm lateral to the beginning of the artery, between the pulmonary cone and the left atrial appendage. Immediately after ligation, the heart was returned to the chest, intrathoracic gas was extruded, and the thoracic cavity was sutured when heart and respiratory rates were stable. The thoracic opening and closing procedure was performed within 30 seconds. The sham group did not undergo ligation surgery. Instead, they only received a thoracotomy and subsequent suturing in the same manner as in the model group. The normal group received no surgery. After surgery, model and sham-operated rats were administered penicillin sodium (80,000 U/rat/day) in order to prevent infection, and indometacin (1 mg/rat/day, 1 mg indomethacin was dissolved in 0.5 ml normal saline) as an analgesic was delivered via intraperitoneal injection for 3 consecutive days following the MI procedure. The control group was given 0.5 ml normal saline intraperitoneally and the sham group was intraperitoneally injected with the same amount of Indomethacin as the MI group.

### Immunohistochemistry

Indoleamine 2,3-dioxygenase expression was evaluated in the hippocampus and distal ileum. Briefly, paraffin sections were dewaxed, blocked with goat serum, and incubated with a rabbit anti-mouse indoleamine 2,3-dioxygenase antibody (1:40, ab106134, Abcam, Cambridge, UK) at 4°C overnight, and subsequently incubated with a goat anti-rabbit secondary antibody (1:1000, ab150077, Abcam, Cambridge, UK) for 30 min at room temperature. Sections were developed for imaging with 3,3'-diaminobenzidine and hematoxylin counterstain. Negative controls were incubated with PBS instead of antibodies. Indoleamine 2,3-dioxygenase-positive cells appeared as brown or yellow-brown particles. Ten fields of vision presenting dense positive particles were selected from the hippocampal CA1 region and distal ileum at low magnification and analyzed using Image-Pro Plus software (Nikon, Tokyo, Japan). Mean absorbance values were measured under high magnification (40X).

### Infarct size measurement

Rats were euthanized and hearts were removed at 4 weeks post-operation. The excised heart was cut into 3 transverse sections and embedded in paraffin. Sections (5-μm thick) were cut. After deparaffinization and dehydration, sections were stained with hematoxylin and eosin (HE) and observed under optical microscopy (CH type, Olympus, Tokyo, Japan). Images were digitized using a computerized image analysis system (Image-Pro Plus 6.0; MediaCybernetics, Japan). Infarct size was calculated by dividing the sum of the planimetered endocardial and epicardial circumferences of the infarcted area by the sum of the total endocardial and epicardial circumferences of the left ventricle. The result was expressed as a percentage [7, 8].

### Behavioral observations

#### Sucrose preference test

On post-operative day 28, a sucrose consumption test was performed as previously described [9, 10, 11]. Rats were single-housed in a quiet room for 2 days and allowed free access to food, tap water, and a 1% sucrose solution. The order of the water and sucrose bottles was changed every day to account for drinking place preferences. The percentage of 1% sucrose consumption within 24 hours was calculated as follows: percentage (%) = total sucrose consumption / (total sucrose consumption + total water consumption) × 100.

#### Forced swim test

On post-operative days 25–26 between the hours of 12:00 and 15:00, the forced swim test was performed as previously described [12, 13]. Briefly, rats were subjected to pre-swimming habituation for 15 minutes on day 25 and tested 24 hours later. Rats were individually placed into Plexiglas cylinders (internal diameter 21 cm; height 50 cm) filled with water at a depth of 30 cm and a temperature of 24–25°C. Rats were forced to swim for consecutive 5 minutes, and the time spent swimming, striving, and immobile and the latency to immobility were analyzed using a swimming analysis system (EthoVision XT, Noldus, the Netherlands).

#### Open-field test

On post-operative days 22–24 between the hours of 13:00 and 16:00, spontaneous behavior and exploration were analyzed using the open-field test as previously described [14, 15, 16]. Briefly, spontaneous behavior was recorded using a detection system (JLBehv-LAR-1; Shanghai Jiliang Software Technology Co., Ltd., Shanghai, China) in a quiet room. The total and central distances explored during the 5-minute test were analyzed.

### Determination of gastric residual and gastrointestinal transit rates

As previously described [17–20], rats were fasted for 24 hours prior to the experiment but allowed free access to water. Each rat was sacrificed after consumption of 1 mL/100 g nutritional black semi-solid paste for 20 minutes. The paste was composed of sodium carboxymethyl cellulose (10 g), milk powder (16 g), sugar (8 g), starch (8 g), and activated carbon (2 g) at 1 mL/100 g. Subsequently, the abdominal cavity was opened, the gastric cardia and pylorus were ligated, and the stomach was harvested, dried, and weighed. The stomach was cut along the greater curvature, the contents were rinsed, and the stomach was weighed again. The difference in gastric weight indicated the weight of the gastric residues. The gastric residual rate was calculated as follows: gastric residual rate (%) = (total gastric weight − gastric net weight) / intragastric volume × 100.

A portion of the small intestine from the pylorus to the ileocecum was cut. The intestinal canal was placed on a glass plate and the carbon propulsion distance and total length of the small intestine were measured using a meter scale. The gastrointestinal transit rate was calculated as follows: intestine propulsion rate (%) = carbon propulsion distance (cm)/total length of small intestine × 100.

### High-performance liquid chromatography with fluorescence and ultraviolet detection

The preparation of plasma samples was as follows: 4 mL abdominal aortic blood samples were collected in 10 mL blood tubes containing heparin. After blood collection, the contents of the tubes were carefully mixed and centrifuged at 4°C for 15 min at 200 g. The resulting platelet-rich plasma (PRP) was transferred into a polypropylene tube and centrifuged at ambient temperature for 20 minutes at 2,000 g. The resulting platelet-poor plasma (PPP) was collected in a polypropylene tube, and a small portion was taken to count the number of platelets in the PPP. The PPP was stored at –80°C until analysis. One hundred μL of PPP was mixed with 100 μL of 5 mmol perchloric acid for 30 seconds and then centrifuged at 17,510 g for 10 minutes at 4°C for detection.

The preparation of brain tissue and ileum samples were as follows and as previously described [16, 17]: hippocampal and ileal specimens were weighed and homogenized in ice-cold 0.5 mmol perchloric acid solution at 3 mL/1 g and 5 mL/1 g, respectively, then immersed in an ice bath for 10 minutes and centrifuged at 5,000 r/min for 10 minutes at 4°C. One hundred μL of homogenate was then mixed with 100 μL of 0.5 mmol perchloric acid solution and centrifuged at 17,510 g for 10 minutes at 4°C. The supernatant was harvested for detection by high performance liquid chromatography with ultraviolet and fluorescence detection (Agilent, USA). Tryptophan and its metabolites were commercially purchased as positive controls (Sigma, USA). The chromatographic column was an Agilent Zorbax Eclipse Plus C18 (4.6 mm × 250 mm, 5 μm). Kynurenine was detected using an ultraviolet detector at 225 nm, whereas 5-HT, tryptophan, 5-hydroxyindole acetic acid, and kynurenic acid were detected using fluorescence at 280 nm, 340 nm, 254 nm, and 404 nm, respectively. Experimental data were analyzed on an Agilent ChemStation (Agilent, USA).

### Statistical analysis

All data were analyzed using SPSS 17.0 software (SPSS, Chicago, IL, USA) and a one-way analysis of variance (ANOVA). All data were subjected to statistical testing for normality and homogeneity of variance before an ANOVA was performed. Differences between the 2 groups were compared using least a significant difference t-test for homogeneity of variances and using Tamhane's T2 method for heterogeneity of variances. All data were expressed as the mean ± SEM. P < 0.05 indicated a statistically significant difference.

## Results

### General information

Of 15 rats in the model group, 8 survived the surgery and 7 died of intraoperative or post-operative arrhythmia, yielding a survival rate of 53.33%. Of 12 rats in the sham group, 2 died of intraoperative arrhythmia and 2 rats died during the recovery period. There were 8 rats in the normal group. Only the 24 surviving rats were included in the final analysis. Rats in the model group exhibited reduced food intake, slower increases in body weight, reduced activity, and nose and limb distal cyanosis post-operation as compared to rats in the sham and normal groups. Temperatures and heart rates did not change appreciably after surgery. There were no signs of lung infection or pneumothorax or pathological state of postoperative inflammation.

### Electrocardiography changes in rats after acute myocardial infarction

**Fig 1:**
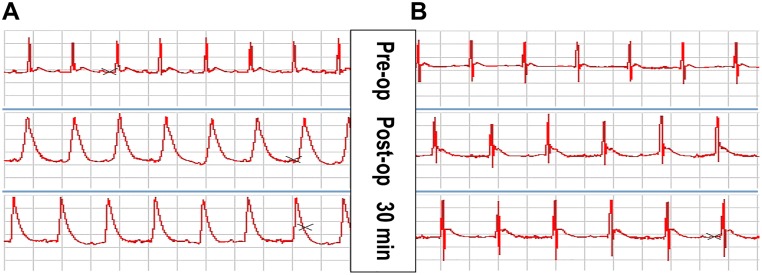
ECG changes at lead II after acute myocardial infarction (AMI) in rats. (A) ECG of the AMI group. The follow-up ECG after AMI shows ST segment elevation at lead II lasting over 30 min. (B) ECG of the Sham group. The follow-up ECG 30 min after surgery was similar to the baseline ECG.

Successful establishment of arterial occlusion was defined by the observation of an ST-segment elevation of 0.2 mV for more than 30 minutes (lead II) and an increased R-wave amplitude on electrocardiography. In the sham group, an ST-segment elevation was not observed post-operation (Fig 1).

### Pathology of cardiac muscle

**Fig 2:**
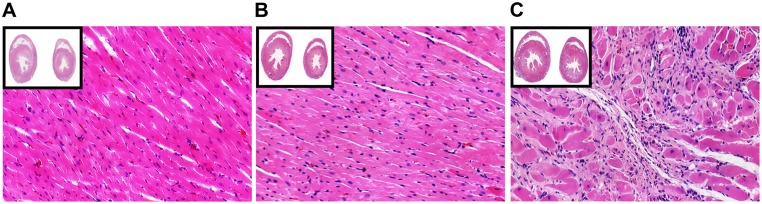
Histopathological changes in heart tissue after acute myocardial infarction (AMI) with hematoxylin-eosin (HE) staining (10X and 20X magnification). (A) HE staining of normal heart tissue. (B) HE staining of heart tissue from the Sham group. (C) HE staining of heart tissue from the AMI group.

In the model group, ventricular wall thinning and collapse, with the infarcted area (38.53 ± 8.39%) showing an ivory-white color, were macroscopically visible at the site of the infarct lesion. In sham-operated rats, the heart was intact and dark red. After hematoxylin-eosin staining, the infarcted cardiac muscle exhibited a large number of structurally disordered fiber tissues, cardiac muscle fiber swelling, absence of muscle striation, and dark eosin staining. Myocardial cell fibrils and some myocardial nuclei were dissolved and absent (colliquative myocytolysis). A large number of infiltrating inflammatory cells was observed and myocardial fibers at the ischemic area were dissolved and ruptured, indicating the proliferation of fibrous tissue. In the normal and sham groups, myocardial cells were arranged in an orderly fashion and exhibited clear nuclei (Fig 2).

### Behavioral observations

#### Sucrose preference test results

**Fig 3:**
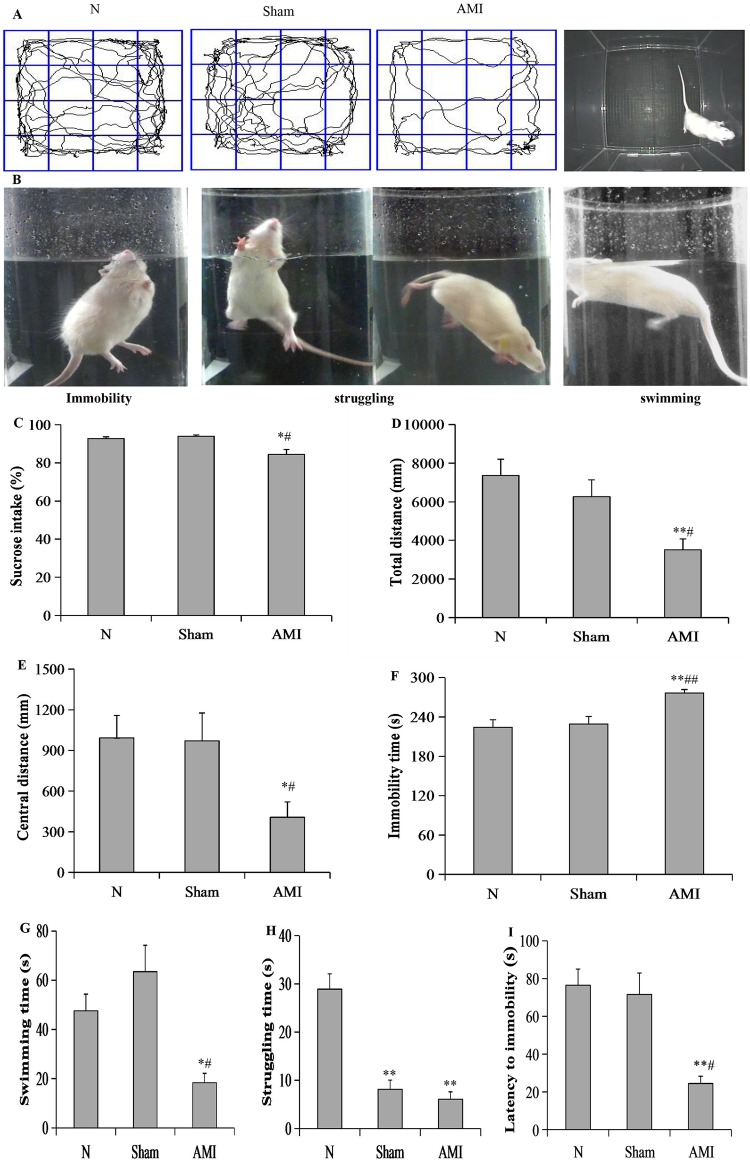
Behavioral measures. (A, B) Representative images of the observed rat behaviors in the open field test (OFT) and forced swim test (FST). (C) Sucrose intake in the 3 groups. (D-E) Total and central distance in the 3 groups. (F-I) Immobility, swimming time, struggling time, and latency to immobility in the 3 groups. Data are presented as means ± S.E.M (n = 8 per group). * P < 0.05 and ** P < 0.01 vs. N group; ^#^ P < 0.05 and ^##^ P < 0.05 vs. Sham group.

A one-way analysis of variance showed significant differences in sucrose intake among the 3 groups at 4 weeks post-operation (F[2, 21] = 9.792; P < 0.01). Further pairwise comparisons showed that sucrose intake in the model group was decreased relative to the sham and normal groups (P = 0.024, P = 0.045, respectively). There were no significant differences between the sham and normal groups (P = 0.736; Fig 3C).

#### Open-field test results

A one-way analysis of variance revealed significant differences in the total and central distance among the 3 groups (F [2, 21] = 6.631, P < 0.01; F [2, 21] = 4.044, P < 0.05, respectively). Further pairwise comparisons showed that the total and central distances were significantly reduced in model group relative to the sham and normal groups (AMI vs. sham, P = 0.019, P = 0.025, respectively; AMI vs. N, P = 0.002, P = 0.020, respectively). There were no significant differences between the sham and normal groups (P = 0.331, P = 0.927, respectively; Fig 3D and 3E).

#### Forced swim test results

A one-way analysis of variance revealed significant differences in immobility time, swimming time, struggling time, and latency to immobility among the 3 groups (F [2, 21] = 8.461, P < 0.01; F [2, 21] = 8.907, P < 0.01; F [2, 21] = 29.434, P < 0.01; F [2, 21] = 11.408, P < 0.01 respectively). Further pairwise comparisons showed that immobility time was prolonged, whereas swimming time and latency to immobility were reduced in the model group relative to the sham and normal groups (AMI vs. sham: P = 0.003, P = 0.011, P = 0.011, respectively; AMI vs. N: P = 0.001, P = 0.01, P = 0.001, respectively). Compared to the normal group, the model and sham groups exhibited reduced struggling time (P = 0.000, P = 0.000, respectively). There were no significant differences in immobility time, swimming time, and latency to immobility between the sham and normal groups (P = 0.713, P = 0.554, P = 0.982, respectively; Fig 3F–3I).

### Analysis of gastric residual and gastrointestinal transit rates

**Fig 4:**
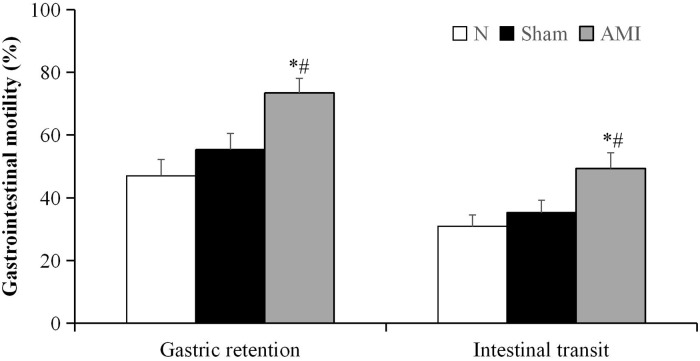
Gastric retention and intestinal transit measured 4 weeks after the acute myocardial infarction (AMI) operation. Data are presented as means ± S.E.M. (n = 8 per group). * P < 0.01 vs. N group; ^#^ P < 0.05 vs. Sham group.

A one-way analysis of variance revealed significant differences in the gastric residual and intestinal transit rates among the 3 groups (F [2, 21] = 7.231, P < 0.01; F [2, 21] = 5.028, P < 0.05, respectively). Further pairwise comparisons showed that the gastric residual and intestinal transit rates were increased in the model group relative to the sham and normal groups (AMI vs. sham, P = 0.019, P = 0.031, respectively; AMI vs. N, P = 0.001, P = 0.006, respectively). There were no significant differences between the sham and normal groups (P = 0.252, P = 0.474; Fig 4).

### Analysis of tryptophan and its metabolites in the rat hippocampus

**Fig 5:**
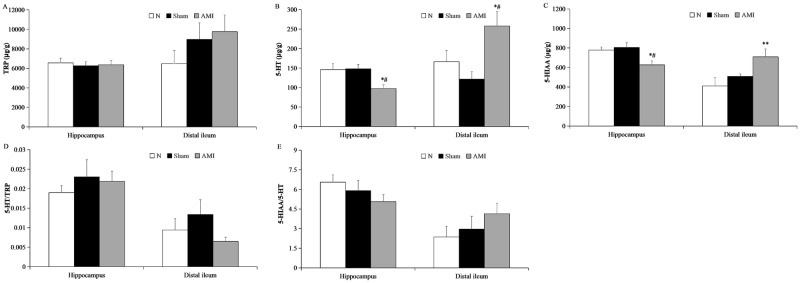
Concentrations of tryptophan (TRP) and its metabolites in the hippocampus and distal ileum. (A-C) Concentrations of TRP, 5-HT, and 5-hydroxyindoleacetic acid (5-HIAA). (D-E) Ratios of 5-HT/TRP and 5-HIAA/5-HT. Data are presented as means ± S.E.M. (n = 8). * P < 0.05 and ** P < 0.01 vs. N group; ^#^ P< 0.01 vs. Sham group.

A one-way analysis of variance showed that there were no significant differences in tryptophan content, the 5-HT/tryptophan ratio, or the 5-hydroxyindoleacetic acid/5-HT ratio in the hippocampal CA1 region among the 3 groups (F [2, 21] = 0.108, P > 0.05; F [2, 21] = 0.441, P > 0.05; F [2, 21] = 1.375, P > 0.05, respectively). However, differences in 5-HT and 5-hydroxyindoleacetic acid content were statistically significantly different among the 3 groups (F [2, 21] = 5.299, P < 0.05; F [2, 21] = 5.483, P < 0.05, respectively). Further pairwise comparisons revealed that hippocampal 5-HT and 5-hydroxyindoleacetic acid content were significantly decreased in the model group relative to the sham and normal groups (AMI vs. sham: P = 0.009, P = 0.006, respectively; AMI vs. N: P = 0.012, P = 0.017, respectively). There were no significant differences between the sham and normal groups (P = 0.893, P = 0.643, respectively; Fig 5).

### Analysis of tryptophan and its metabolites in the distal ileum

A one-way analysis of variance revealed that there were no significant differences in tryptophan content, 5-HT/tryptophan ratio, or 5-hydroxyindoleacetic acid/5-HT ratio in the distal ileum among the 3 groups (F [2, 21] = 1.138, P > 0.05; F [2, 21] = 1.475, P > 0.05; F [2, 21] = 1.083, P > 0.05, respectively). However, differences in 5-HT and 5-hydroxyindoleacetic acid content were statistically significant among the 3 groups (F [2, 21] = 5.579, P < 0.05; F [2, 21] = 4.802, P < 0.05, respectively). Further pairwise comparisons showed that 5-HT content in the model group was increased relative to the sham and normal groups (P = 0.004, P = 0.039, respectively), but no difference was found between the sham and normal groups (P = 0.296). Compared to the normal group, 5-hydroxyindoleacetic acid content was increased in the model group (P = 0.006, Fig 5).

### Analysis of tryptophan and its metabolites in plasma

**Fig 6:**
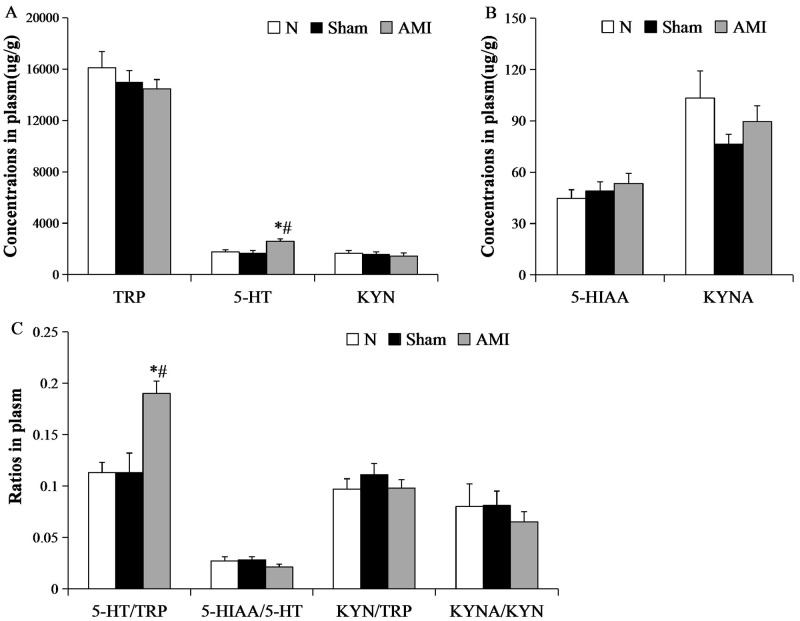
Concentrations of TRP and its metabolites in the plasma using HPLC. (A) Concentrations of (A) tryptophan (TRP), 5-HT, and kynurenine (KYN); (B) 5-hydroxyindoleacetic acid (5-HIAA) and kynurenic acid (KYNA); (C) the ratios of 5-HT/TRP, 5-HIAA/5-HT, KYN/TRP, KYNA/KYN. Each bar represents mean ± S.E.M. of each group of mice (n = 8 per group). * P < 0.01 vs. N group; ^#^ P < 0.01 vs. Sham group.

A one-way analysis of variance showed that there were no significant differences in plasma tryptophan, 5-hydroxyindoleacetic acid, kynurenine, or kynurenic acid content, and furthermore there were no significant differences in 5-hydroxyindoleacetic acid/5-HT ratio, kynurenine/tryptophan ratio, or kynurenic acid/kynurenine ratio among the 3 groups (F [2, 21] = 0.727, P > 0.05; F [2, 21] = 0.653, P > 0.05; F [2, 21] = 0.282, P > 0.05; F [2, 21] = 1.469, P > 0.05; F [2, 21] = 1.400, P > 0.05; F [2, 21] = 0.659, P > 0.05; F [2, 21] = 0.310, P > 0.05, respectively). 5-HT content and 5-HT/tryptophan ratio were significantly different among the 3 groups (F [2, 21] = 7.652, P < 0.01; F [2, 21] = 9.920, P < 0.01, respectively). Further pairwise comparisons showed that serum 5-HT content and the 5-HT/tryptophan ratio in the model group were increased relative to the sham and normal groups (AMI vs. sham: P = 0.002, P = 0.001; AMI vs. N: P = 0.005; P = 0.001), whereas there were no significant differences between the sham and normal groups (P = 0.684, P = 0.969, respectively; Fig 6).

### Immunohistochemical detection of indoleamine 2,3-dioxygenase expression in the hippocampal CA1 region and the terminal ileum

**Fig 7:**
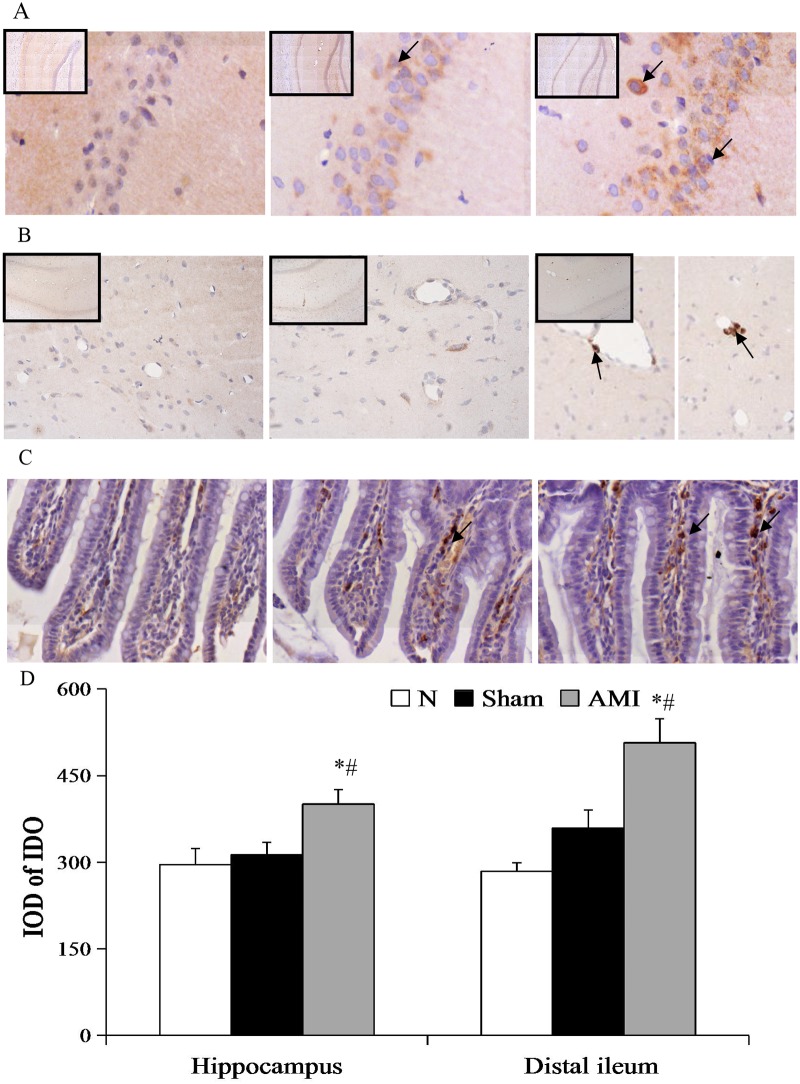
Photomicrographs of indoleamine 2,3-dioxygenase (IDO) in the hippocampus and distal ileum. Arrows indicate IDO-positive cells. (A) The expression of IDO in the hippocampus CA1 region (magnification 10X, 20X), and (B) microvasculature (magnification 5X, 20X). (C) The expression of IDO in the distal ileum (magnification 40X). (D) The expression of IDO in the hippocampus CA1 region and distal ileum. Data are presented as means ± S.E.M. (n = 10). * P < 0.01 vs. N group; ^#^ P < 0.05 vs. Sham group.

A one-way analysis of variance showed significant differences in indoleamine 2,3-dioxygenase expression in the hippocampal CA1 region and the distal ileum among the 3 groups (F [2, 27] = 5.127, P < 0.05; F [2, 27] = 13.046, P < 0.01, respectively). Further pairwise comparisons showed that indoleamine 2,3-dioxygenase expression in the hippocampus and the distal ileum was significantly increased in the model group relative to the sham and normal groups (AMI vs. sham: P = 0.019, P = 0.035; AMI vs. N: P = 0.006, P = 0.001), whereas there were no significant differences between the sham and normal groups (P = 0.636, P = 0.144; Fig 7).

## Discussion

Myocardial infarction is characterized by high rates of morbidity and mortality and is often comorbid with depression and gastrointestinal dysfunction [1–5]. The central brain regulates memory, mood, and behavior, and plays a crucial role in psychological and somatic disease through connections between the hippocampus, amygdala and prefrontal cortex, and amygdala and cingulate cortex [6, 9, 10, 20]. 5-HT is an important neurotransmitter that regulates emotional status and depression. The 5-HT hypothesis of the pathogenesis of depression was first proposed in 1969 [21]. In accordance with this hypothesis, tryptophan metabolic disorders and tryptophan shunts can increase kynurenine synthesis and decrease 5-HT synthesis, which is a major cause of depression. The inflammation hypothesis of depression also supports the 5-HT hypothesis, as it highlights the involvement of increased expression of indoleamine 2,3-dioxygenase (a rate-limiting enzyme in the tryptophan-kynurenine metabolic pathway) and posits the inhibition of 5-HT reuptake would be effective for the treatment of depression [22].

### Depression after myocardial infarction and hippocampal 5-HT metabolic abnormalities

Accumulating evidence indicates that the decline of hippocampal 5-HT release is highly correlated with the presentation of depressive disorder [23, 24]. Tryptophan is a 5-HT precursor and approximately 95% of tryptophan metabolism is mediated by indoleamine 2,3-dioxygenase or tryptophan 2,3-dioxygenase via the kynurenine pathway [25, 26]. Kynurenine prevents tryptophan from crossing the blood-brain barrier and promotes indoleamine 2,3-dioxygenase activity [27, 28], which decreases the production of 5-HT and can lead to the development of depression. While kynurenic acid generated via kynurenine metabolic pathways is the only known endogenous antagonist of the N-methyl-DL-aspartate receptor and can function to prevent depression, kynurenic acid has a greater affinity for alpha-7-nicotinic acetylcholine receptors, which have been implicated in depression, dementia, and other mental disorders [29, 30]. This evidence suggests that indoleamine 2,3-dioxygenase may activate the tryptophan-kynurenine pathway, leading to abnormal 5-HT levels in the brain and depressive disorders.

We evaluated depressive-like behavior in rats after myocardial infarction. Depressive-like behavior is a complex disorder presenting with a lower degree of curiosity about novelty, decreased exploration capacity, lack of interest, loss of pleasant feelings, and despair. In this study, the open field test was used to assess exploratory activity, which is a common method to test the depression-like and anxiety-like behavior. The lack of exploratory behavior can occur in both depression and anxiety. However, in depression, in addition to the lack of exploratory behavior, lack of pleasure and helplessness are also characteristic manifestations. In contrast, in anxiety, besides the lack of exploratory behavior, increased fear, micturition, defecation, or reduced social behavior is involved. In our present study, the open field test, forced swimming test, and sucrose preference test were used together to assess depressive-like behavior and reflect typical depression performance. We found that, after myocardial infarction, sucrose preference was significantly decreased and the total and central distance traveled in the open field test were significantly decreased. Additionally, immobility time in the forced swimming test was increased, and swimming time and latency to immobility were shortened. These behaviors are supportive of depressive disorder after myocardial infarction, consistent with behavioral performance in other depression models [31]. Changes in 5-HT were assayed by high performance liquid chromatography with fluorescence and ultraviolet detection. 5-HT and 5-hydroxyindole acetic acid in hippocampal tissues were significantly decreased in rats post-myocardial infarction, suggesting a reduction in the conversion of 5-HT to 5-HIAA. These data are consistent with the 5-HT hypothesis of depression. Our experimental findings suggest that depression after myocardial infarction has a similar biological basis as depression not associated with myocardial infarction.

It has been proposed that the synthesis of 5-HT in the brain is influenced by tryptophan levels in peripheral blood [32, 33]. Tryptophan and other neutral amino acids competitively bind to transporters and cross the blood-brain barrier to exit the central compartment. Sharp decreases in the level of tryptophan in the brain ultimately lead to decreases in 5-HT synthesis. In this study, hippocampal tryptophan content in the model group was not different from that in the sham or normal groups, suggesting that after tryptophan crossed the blood-brain barrier, 5-HT and the generation of its degradation product 5-HIAA decreased. This result is not in agreement with the peripheral blood tryptophan depletion theory. Rather, in this study, indoleamine 2,3-dioxygenase expression was upregulated in the hippocampus, suggesting abnormalities in tryptophan metabolism via the kynurenine pathway. The reduced conversion of 5-HT to 5-HIAA is a possible mechanism underlying low levels of hippocampal 5-HT. At present, hippocampal 5-HT metabolism and indoleamine 2,3-dioxygenase expression after myocardial infarction are not well studied. Our experimental findings indicate that low metabolism of 5-HT is associated with indoleamine 2,3-dioxygenase activation in the hippocampus, potentially in the microglia. This speculation is in agreement with a previous study that showed that the increased activity of indoleamine 2,3-dioxygenase leads to decreased hippocampal 5-HT in chronic stress-exposed depressive rats [34].

### Gastrointestinal dysfunction and 5-HT metabolic abnormalities after myocardial infarction

5-HT is also an important neurotransmitter in the enteric nervous system and functions to regulate intestinal secretion and motility [35]. In humans, 95% of 5-HT is synthesized in the intestinal tract and 90% is distributed to intestinal chromaffin cells, whereas the remaining 5% is found in the enteric nervous system [36]. During stress, the enteric cavity stimulates the secretion of 5-HT, which acts on intrinsic primary afferent neurons in the mucosal membrane. These afferent neurons can activate interneurons in the dorsal horn and initiate intestinal tract movement through receptor pathways [37–39]. Under pathological conditions, increases in 5-HT play a role in gastrointestinal motility dysfunction.

In this experiment, rats exhibited poor appetite and severe flatulence after myocardial infarction. Gastric residual and intestinal propulsion rates were consistent with gastric retention and bowel irritation, indicating a gastrointestinal motility disorder. At the same time, ileal 5-HT content, as detected by high performance liquid chromatography, was increased, suggesting that 5-HT elevations are involved in gastrointestinal dysfunction after myocardial infarction. The mechanism of this relationship may be as follows: (1) ileal 5-HT and its downstream product 5-hydroxyindoleacetic acid were significantly increased as a result of increased 5-HT synthesis, rather than 5-HT metabolic dysfunction; (2) indoleamine 2,3-dioxygenase expression was increased in the ileal epithelium and lamina propria due to increased 5-HT content in the intestinal tract. Indoleamine 2,3-dioxygenase expression is promoted by pro-inflammatory cytokines such as IFN-α, TNF-α, and IL-1β [40, 41]. 5-HT in the intestinal mucosa has anti-inflammatory effects, and 5-HT in intestinal nerves both inhibits inflammatory nerve damage and activates 5-HT_4_ receptors to promote nerve regeneration. Through the aforementioned effects, 5-HT has neuroprotective roles, in addition to regulatory functions, in the intestine. Thus, inflammation is associated with high 5-HT metabolism (5-HT to 5-HIAA). In Crohn's disease and ulcerative colitis, increased expression of indoleamine 2,3-dioxygenase is associated with gastrointestinal dysfunction [42] and in irritable bowel syndrome, a simultaneous reduction of kynurenic acid and 5-HT in the duodenal mucosa is observed [43, 44]. In general, as 5-HT levels and the conversion of tryptophan to kynurenic acid are increased, tryptophan levels should decrease. However, in our study, we observed no changes in intestinal tryptophan. According to the literature [45], tryptophan accumulates in the intestinal mucosa under conditions of oxidative stress. Therefore, we speculate that the tryptophan levels we observed in the intestine were the simultaneous consequence of tryptophan metabolism to kynurenic acid and oxidative stress-related generation of tryptophan. This suggests that tryptophan-to-5-HT and tryptophan-to-kynurenic acid metabolism in the intestine differ from those in hippocampal tissue and do not conform to the 5-HT hypothesis.

### Gastrointestinal dysfunction and depression after myocardial infarction

Patients with depression after myocardial infarction may present with both cognitive/psychiatric symptoms and somatic/psychiatric symptoms. It is known that somatic/psychiatric symptoms are independent factors that affect the disease prognosis [46, 47]. However, the pathogenesis of depression after myocardial infarction remains elusive, and it is unclear whether depression occurs as an effect of the disease or as an independent clinical syndrome. There are two different bases for the association between nervous system and gastrointestinal tract diseases, namely the "brain-gut axis" and the "gut-brain axis". The brain-gut axis refers to the idea that the gut is regulated by the central nervous system, whereas the gut-brain axis refers to the concept that brain function and behavior are affected by gut-derived cytokines, hormones, and microbes [48–50]. These regulatory axes are closely linked but functionally distinct.

In this study, 5-HT content was decreased in the hippocampus but increased in the intestinal tract and peripheral blood after myocardial infarction. As 5-HT in peripheral blood is primarily derived from the intestinal tract, increased 5-HT in peripheral blood may have been a result of increased 5-HT secretion in the intestinal tract. This hypothesis is consistent with the observation of increased 5-HT in peripheral blood in a model of stress-induced depression [51]. Because 5-HT in peripheral blood has a vasoconstrictor effect [52], it may alter vascular permeability in damaged vascular endothelial cells and induce vasospasm [53], subsequently aggravating ischemia/hypoxia and inflammatory reactions in the intestinal tract and hippocampus. Inflammatory reactions may then promote indoleamine 2,3-dioxygenase expression and the metabolism of elevated 5-HT in the intestine, leading to a cycle of abnormal gastrointestinal motility, gastric distention, and loss of appetite. Furthermore, increases in the expression of indoleamine 2,3-dioxygenase in the hippocampus may also underlie decreases in 5-HT and depressive behavior after myocardial infarction.

Although we draw some meaningful results, some limitations must be mentioned. In this article, we confirmed abnormal 5-HT metabolism and IDO expression in myocardial infarction rats, but there is no direct evidence for the relationship between 5-HT metabolic pathways and IDO. Besides, whether tryptophan deprivation or inhibiting 5-HT pathway or blocking IDO can affect depression and gastrointestinal dysfunction after myocardial infarction, still needs further study.

## Conclusion

In summary, we conclude that after myocardial infarction, intestinal hypoperfusion produced ischemia and hypoxia that increased the synthesis of intestinal 5-HT and the release of 5-HT into peripheral blood. Subsequently, increased levels of 5-HT on the vascular endothelium led to vasoconstriction and permeability changes that exacerbated impaired bowel functions, thereby initiating a damaging cycle. Our experimental findings indicate that the inverse relationship between the level of 5-HT in peripheral blood and the level of 5-HT in the hippocampus is related to high metabolism of 5-HT in the intestine S1 Supporting Information. 5-HT in peripheral blood serves as a bridge between the gut and the brain and is an important regulator of the gut-brain and brain-gut axes. Abnormal 5-HT metabolism in the intestinal tract plays a crucial role in the presentation of depression after myocardial infarction and may serve as the biological basis for somatic/psychiatric symptoms.

5-HT is an essential neurotransmitter for information transmission in the central and enteric nervous systems. We observed that abnormalities in 5-HT metabolism are involved in the onset of depression and gastrointestinal dysfunction after myocardial infarction. Specifically, we observed abnormalities in the tryptophan-to-5-HT metabolic pathway. This evidence suggests that somatic/psychiatric symptoms and cognitive/psychiatric symptoms have a common biological basis after myocardial infarction. Understanding the pathophysiology of myocardial infarction and its relationship with comorbid disorders is vital for assessing disease prognosis and improving the therapeutic approach.

## Supporting information

S1 Supporting Information All the original data of our manuscript. The original data (Mean and Standard error of each value) of Figs 3–7 was included.
(XLS)
